# Expansion of Regulatory T Cells in Patients with Langerhans Cell Histiocytosis

**DOI:** 10.1371/journal.pmed.0040253

**Published:** 2007-08-14

**Authors:** Brigitte Senechal, Gaelle Elain, Eric Jeziorski, Virginie Grondin, Natacha Patey-Mariaud de Serre, Francis Jaubert, Kheira Beldjord, Arielle Lellouch, Christophe Glorion, Michel Zerah, Pierre Mary, Mohammed Barkaoui, Jean Francois Emile, Liliane Boccon-Gibod, Patrice Josset, Marianne Debré, Alain Fischer, Jean Donadieu, Frederic Geissmann

**Affiliations:** 1 INSERM, U838, Laboratory of Biology of the Mononuclear Phagocyte System, Necker Enfants Malades Institute, Paris, France; 2 Université Paris Descartes Medical School, Paris, France; 3 Department of Pathology, Hôpital Necker Enfants Malades and Assistance Publique–Hôpitaux de Paris, Paris, France; 4 Department of Laboratory Medicine, Hôpital Necker Enfants Malades and Assistance Publique–Hôpitaux de Paris, Paris, France; 5 Department of Orthopedic Surgery, Hôpital Necker Enfants Malades and Assistance Publique–Hôpitaux de Paris, Paris, France; 6 Department of Neurosurgery, Hôpital Necker Enfants Malades and Assistance Publique–Hôpitaux de Paris, Paris, France; 7 Department of Orthopedic Surgery, Hôpital d'Enfants Armand Trousseau and Assistance Publique–Hôpitaux de Paris, Paris, France; 8 Delegation a la Recherche Clinique, Centre Hospitalier Universitaire de Nantes, Nantes, France; 9 Laboratory of Pathology, Hôpital Ambroise-Paré and Assistance Publique–Hôpitaux de Paris Boulogne Billancourt, France; 10 Department of Pathology, Assistance Publique–Hôpitaux de Paris, Hôpital d'Enfants Armand Trousseau, Paris, France; 11 Immunology and Hematology Unit, Department of Pediatrics, Assistance Publique–Hôpitaux de Paris and Necker Enfants Malades Hospital, Paris, France; 12 Department of Hematology, Assistance Publique–Hôpitaux de Paris and Hôpital d'Enfants Armand Trousseau, Paris, France; Centre d'Immunologie, France

## Abstract

**Background:**

Langerhans cell histiocytosis (LCH) is a rare clonal granulomatous disease that affects mainly children. LCH can involve various tissues such as bone, skin, lung, bone marrow, lymph nodes, and the central nervous system, and is frequently responsible for functional sequelae. The pathophysiology of LCH is unclear, but the uncontrolled proliferation of Langerhans cells (LCs) is believed to be the primary event in the formation of granulomas. The present study was designed to further investigate the nature of proliferating cells and the immune mechanisms involved in the LCH granulomas.

**Methods and Findings:**

Biopsies (*n* = 24) and/or blood samples (*n* = 25) from 40 patients aged 0.25 to 13 y (mean 7.8 y), were studied to identify cells that proliferate in blood and granulomas. We found that the proliferating index of LCs was low (∼1.9%), and we did not observe expansion of a monocyte or dendritic cell compartment in patients. We found that LCH lesions were a site of active inflammation, tissue remodeling, and neo-angiogenesis, and the majority of proliferating cells were endothelial cells, fibroblasts, and polyclonal T lymphocytes. Within granulomas, interleukin 10 was abundant, LCs expressed the TNF receptor family member RANK, and CD4^+^ CD25^high^ FoxP3^high^ regulatory T cells (T-regs) represented 20% of T cells, and were found in close contact with LCs. FoxP3^+^ T-regs were also expanded compared to controls, in the blood of LCH patients with active disease, among whom seven out of seven tested exhibited an impaired skin delayed-type hypersensitivity response. In contrast, the number of blood T-regs were normal after remission of LCH.

**Conclusions:**

These findings indicate that LC accumulation in LCH results from survival rather than uncontrolled proliferation, and is associated with the expansion of T-regs. These data suggest that LCs may be involved in the expansion of T-regs in vivo, resulting in the failure of the host immune system to eliminate LCH cells. Thus T-regs could be a therapeutic target in LCH.

## Abstract 2

Frederic Geissmann and colleagues find that Langerhans cell accumulation in Langerhans cell histiocytosis results from survival rather than uncontrolled proliferation, and is associated with the expansion of regulatory T cells.

## Editors' Summary

**Background.:**

Langerhans cell histiocytosis (LCH) is a rare disease, affecting mainly children, in which the number of Langerhans cells (immune system cells that are also known as histiocytes) in the body greatly increases. In LCH, immature Langerhans cells spread throughout the body—they are usually found only in the skin and airways—and accumulate in small inflamed nodules called granulomas. The symptoms and severity of LCH depend on where these granulomas (which contain several different types of cells) occur. Granulomas in bone, for example, can weaken the bone and lead to frequent fractures. Other symptoms of LCH include skin rashes, breathing difficulties, and hearing problems. LCH is usually treated with corticosteroids, drugs that suppress immune function, but if the disease is widespread, anticancer drugs may also be used. Most affected children recover from the disease but the disease can be fatal if multiple organs are affected.

**Why Was This Study Done?:**

For many years LCH has been regarded as a cancer-like condition (hence the use of anticancer drugs in its treatment) in which the uncontrolled proliferation of Langerhans cells drives the formation of granulomas. However, some researchers are beginning to ask whether LCH might actually be a problem with the immune system—Langerhans cells are dendritic cells, and these normally activate the immune response when the body is challenged by bacteria or viruses. To find better ways to treat LCH It is important to understand the underlying defect in the disease and how it develops. In this study, the researchers have investigated which cells in LCH granulomas are proliferating and whether immune mechanisms are involved in the development of LCH.

**What Did the Researchers Do and Find?:**

The researchers stained slices of LCH granulomas with antibodies (proteins made by the immune system) that label different types of cell and with an antibody that recognizes Ki-67, a protein made by proliferating cells. On average, only 6% of the proliferating cells in the granulomas were Langerhans cells. 12% were T lymphocytes (immune system cells that directly kill bacteria and viruses and stimulate antibody production by B lymphocytes). The rest were endothelial cells (which line blood vessels) and fibroblasts (which form the framework that supports the tissues of the body). These data suggest that abnormal proliferation of Langerhans cells is not responsible for maintenance and spread of granulomas—so could increased survival of these cells lead to their accumulation in granulomas instead? When the researchers investigated the immunological characteristics of LCH granulomas, they found that many of the T cells in the granulomas were regulatory T cells (T-regs), a type of T cell that inhibits T cell responses and prevents the body from attacking itself. Consistent with the presence of T-regs, IL10 (a protein made by T cells that suppresses the function of Langerhans cells) was abundant within the granulomas. Furthermore, the Langerhans cells in the granulomas expressed RANK, a protein that stimulates the proliferation of T-regs, and patients with LCH had a higher proportion of T-regs in their blood than did healthy children; this proportion decreased during their treatment for LCH. Finally, all the children with LCH that the researchers tested had an impaired delayed-type hypersensitivity response, an indication that the increased numbers of T-regs had inhibited their immune system.

**What Do These Findings Mean?:**

These findings indicate that proliferation of Langerhans cells is not the driving force behind the development of LCH. Instead they suggest that abnormal Langerhans cells induce the accumulation of T-regs within the granuloma. These T-regs inhibit the induction of an efficient immune response against the Langerhans cells, and this sets up a vicious cycle in which the increased survival of Langerhans cells leads to their accumulation in granulomas, the production of more T-regs, and so on. This new model, although it is based on the investigation of a small number of patients with LCH, strongly suggests that drugs that target T-regs should be investigated as treatments for LCH.

**Additional Information.:**

Please access these Web sites via the online version of this summary at http://dx.doi.org/10.1371/journal.pmed.0040253.

- Information is available for patients and health professionals on Langerhans cell histiocytosis from the Histiocytosis Association of America
- Information is also available (in French) for patients and health professionals on the Histiocytose.org Web site, which has links to the Association Histiocytose France and Groupe d'Etude des Histiocytoses
- The MedlinePlus encyclopedia contains a page on histiocytosis (in English and Spanish)
- Information is made available by the UK charity Cancerbackup on Langerhans cell histiocytosis in children
- Wikipedia page on regulatory T cells (note: Wikipedia is a free online encyclopedia that anyone can edit; available in several languages)

## Introduction

Langerhans cell histiocytosis (LCH), also known as histiocytosis X, affects mainly young children with a peak incidence between the ages of 1 and 3 y, and features granulomas consisting of macrophages, multinucleated giant cells, lymphocytes, eosinophils, and CD1a^+^ Langerin^+^ Langerhans-like cells, accumulating within various tissues such as bone, skin, lung, liver, bone marrow, lymph nodes, the gastrointestinal tract, and the central nervous system [1–6]. The clinical course of LCH is remarkably varied, ranging from lesions that spontaneously resolve, to a chronic disease, or even to a widespread and sometimes lethal disease [1,7,8]. The pathophysiology of LCH remains enigmatic, but seems to be associated with abnormalities in the biology of Langerhans cells (LCs) and macrophages [5,9,10]. Genetic factors are suspected because of the existence of familial cases [11–13], suggesting that predisposing mutations could be present at least in some patients. Serum cytokines that may promote the growth of dendritic cells (DCs), such as GM-CSF (granulocyte macrophage-colony stimulating factor), M-CSF (macrophage-colony stimulating factor), and FLT3-L (FMS-like tyrosin kinase 3 ligand), have been shown to be increased in the blood of patients with the more severe form of the disease [9,14]. LCs have been shown to be clonal by X-inactivation studies [15,16] (reviewed in [10,17]), and proliferation of LCs has been generally proposed as the mechanism responsible for LC accumulation in most [3,16,18,19], but not all [20] studies. Cytogenetic abnormalities such as loss of heterozygosity of tumor suppressor genes and chromosomal instability have also been described as case reports [21–23], although no recurrent molecular abnormality has been yet characterized. Active viral infections such as herpesvirus infections may worsen the disease [24]; a possible causative role of herpesviruses in LCH has been debated [25–28].

LCs are members of the DC family, which triggers and shapes immune responses, and the pathophysiology of LCH is likely to involve immune mechanisms (reviewed in [10,17]). We have previously reported that LCs found in LCH granulomas were functional semi-mature LCs, able to regulate T cell proliferation [5]. The present study was designed to further investigate the nature of proliferating cells and the immune mechanisms involved in LCH granulomas.

## Materials and Methods

### Patients

Fresh and/or frozen biopsies from granulomas, and/or peripheral blood specimens from 40 pediatric patients with LCH (Table 1) were obtained after written, witnessed, informed consent was obtained from the parents of all patients. All patients were included in the French LCH registry and the research protocol was approved by the ethics committee of the Centre Hospitalier Universitaire de Nantes, France. The inclusion criteria as well as the definition of the organs involved for the French nationwide LCH survey have been described elsewhere [1]. The status of the disease and time of specimen collection relative to treatments are given in the Table 1. LCH tissues (patients 1–12, 29, and 35–37) and peripheral blood mononuclear cells (PBMCs) (patients 3 and 12–34) were obtained at the time of diagnosis and before treatment except otherwise indicated. To study the tuberculin PPD-skin reaction in patients previously vaccinated with BCG (Table 2), 10 TU of PPD (Pasteur-Merieux, http://www.sanofipasteur.com/) was injected intradermally in the forearm of patients. The result was read 3 d later and any induration of 6 mm or more, when read across the forearm, was regarded as a positive result [29], an induration of 4–5.9 mm as a weak positive, and an induration less than 4 mm as a PPD-negative.

**Table 1:**
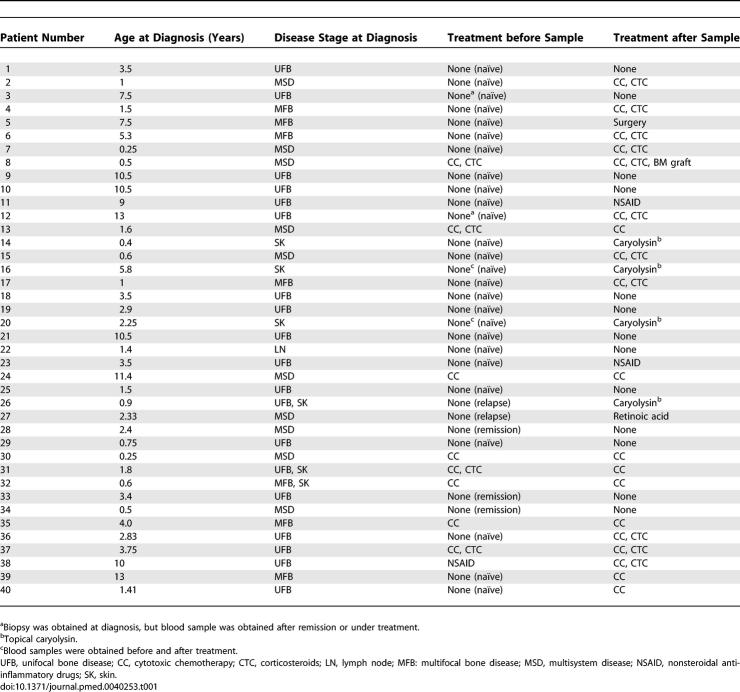
Patient Characteristics

**Table 2:**
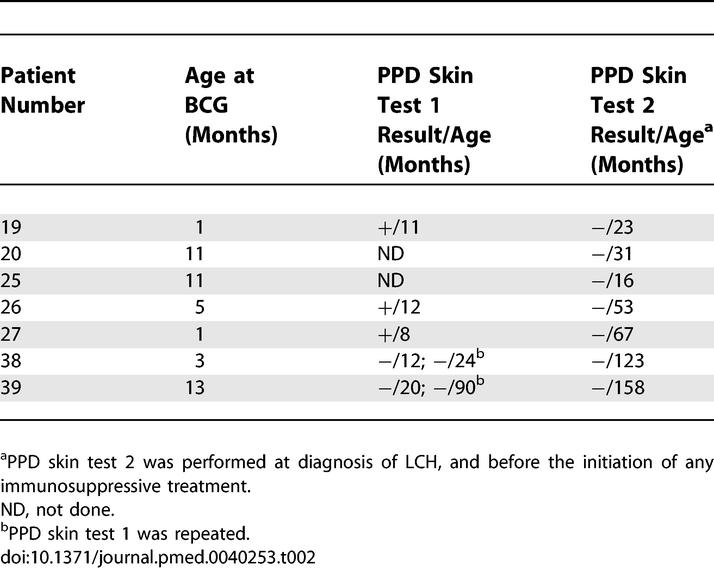
PPD Results in Tested Patients

Biopsies and peripheral blood specimens were also obtained from four pediatric patients with sinus hyperplasia with massive lymphadenopathy (SHML, or Rosai-Dorfman Disease) after written, witnessed, informed consent was obtained from the parents of all patients.

### Statistical Methods

Stata Software (http://www.stata.com) version 8 was used for statistical analyses. Quantitative data were studied by the Kruskal-Wallis test. All tests were two-tailed. *p*-Values of less than 0.05 were considered to indicate statistical significance.

### Immunohistochemistry

For proliferation studies, paraffin-embedded and cryostat sections of biopsy specimens were examined by immunohistochemistry. Cryostats sections were fixed with acetone before staining. Paraffin-embedded sections were heat-treated in a target retrieval solution (pH 6, Dako, http://www.dako.com) for 20 min at 95 °C.

Double staining was performed with the double stain EnVision kit (Dako) according to the manufacturer's instructions. Sections were labeled with mouse anti-Ki-67 (clone MIB-1 1:100, Dako) revealed with horseradish peroxidase anti-mouse antibody and diaminobenzidine substrate and then labeled with mouse anti-CD1a (clone O10 1:2, Beckman Coulter Immunotech, http://www.beckmancoulter.com/), rabbit polyclonal anti-CD3 (A0452 1:100, Dako), mouse anti-CD68 (clone KP1 1:3,000, Dako), or mouse anti_CD20 (L26 1:200, Dako), revealed by alkaline phosphatase anti-mouse/rabbit antibody and Fast blue substrate. Paraffin-embedded sections were labeled with mouse anti-CD1a, and detected with horseradish peroxidase anti-mouse antibody and diaminobenzidine substrate, then labeled with mouse anti-RANK (clone 80707 1:500, R&D Systems, http://www.rndsystems.com/) and detected by alkaline phosphatase anti-mouse/rabbit antibody and Fast blue substrate. For each biopsy a minimum of 100 cells were counted from at least three distinct microscopic fields randomly chosen and photographed.

Sections of formalin-fixed paraffin embedded biopsy were examined by immunohistochemistry for expression of FoxP3 using a mouse monoclonal anti-human FoxP3 antibody (clone 236A/E7 1:40, #Ab20034, Abcam, http://www.abcam.com), endothelial cell markers with mouse anti-CD34 (clone Qbend 10 1:800, Beckman Coulter Immunotech) and mouse anti-CD31 (clone JC70A 1:20, Dako), and TRANCE with mouse anti-RANKL (clone 70525, R&D Systems). Primary antibodies were revealed with a biotinylated secondary antibody, streptavidin peroxidase and diaminobenzidine as a substrate (ChemMate detection kit, Dako) [5]. Sections were then counterstained with hematoxylin.

### Immunofluorescence

For staining of FoxP3 on cryostat sections, sections were incubated with a polyclonal rabbit anti-FoxP3 1:100 (Ab10563, lot 79423, Abcam, Cambridge, UK) for 1 h at 37 °C. Secondary antibodies were biotinylated donkey anti-rabbit (Jackson Immunoresearch, http://www.jacksonimmuno.com/) and Alexa 488-conjugated streptavidin. For CD3, CD25, Ki-67, and CD1a staining, mouse anti-CD3 (clone UCHT1 1:50, Dako), mouse anti-CD25 (clone 2A3 1:20, BD Biosciences), mouse anti-Ki-67 (clone MIB-1 1:100, Dako), and mouse anti-CD1a (clone O10 1:2, Beckman Coulter Immunotech) were revealed by Cy3 anti-mouse antibodies. DAPI or TOPRO-3 was used to stain nuclei. DAPI-stained sections were examined with an inverted fluorescence microscope (Zeiss) coupled to a Coolsnap ES video camera (Roper Scientific, http://www.roperscientific.com/), and the images were analyzed using Meta Morph software (Universal Imaging, http://www.photomet.co.uk/). TOPRO-3-stained sections were examined with an Axiovert 200 attached to LSM 510 confocal system was use to visualize TOPRO-3. For each biopsy a minimum of 100 cells were counted from three to five distinct microscopic fields randomly chosen and photographed.

### Isolation of Mononuclear Cells from Fresh Biopsies

Sterile tissues from eosinophilic granulomas were harvested in RPMI and digested in the presence of collagenase D (1 mg/ml) for 45 min at 37 °C. Cell suspensions were then passed through a 70 μm cell strainer, and mononuclear cells were separated over Ficoll-Paque Plus (Amersham, http://www5.amershambiosciences.com/).

### Flow Cytometry

Mononuclear cells either from fresh biopsies (patients 11, 12, and 30) or peripheral blood (patients 3 and 12–34) were stained with antibodies against CD3 (pacific blue, clone UCHT1) from Dako; CD4 (FITC, clone Leu 3a + 3b), CD25 (PE, clone 2A3), CD8 (APC, clone RPA-T8), HLA-DR (biotinylated, clone L243), CD14 (PE, clone M5E2), CD16 (PE/FITC, clone 3G8), CD11c (APC, clone S-HCL-3), from Becton Dickinson; CD20 (PE, clone 2H7), from eBioscience (http://www.ebioscience.com/); CD123 (APC, cloneAC145), BDCA-1 (FITC, cloneAD5-8E7), BDCA-2 (FITC, clone AC144), from Miltenyi Biotech (http://www.miltenyibiotec.com/). Lineage negative (lin^−^) antibodies to detect myeloid DCs (MDCs) included PE-CD19, PE-CD16, and PE-CD14. Cascade Blue-streptavidin from Molecular Probes/Invitrogen (http://probes.invitrogen.com/) stained cells marked with biotinylated antibodies.

APC anti-human FoxP3 (clone PCH101) staining kit from eBioscience was used after CD3, CD4, and CD25 cell surface staining according to the manufacturers instructions to detect regulatory T cells (T-regs).

Stained cells were acquired with a three-laser, nine-color CyanADP flow cytometer and analyzed with Summit 4.1 software (Dako).

### T Cell Isolation

T cells were isolated either from biopsies or from PBMCs using CD3^+^ selection (Miltenyi Biotec) according to the manufacturer's instructions. CD3^+^ cell purity was controlled by flow cytometry and was >95%. For stimulation with soluble OKT3, CD14^+^ monocytes were positively selected from the same healthy blood donor using CD14-microbeads (Miltenyi Biotec) with a high purity (>95%). CD4^+^ CD25^hi^ and CD4^+^ CD25^lo^ T cells were purified from LCH peripheral blood using the “CD4^+^ CD25^+^ regulatory T cell isolation kit” (Miltenyi Biotec). Each fraction's purity was over 75%.

### T Cell Proliferation Assay

CD3^+^ T cells were purified from fresh biopsies (LCH T cells) and from healthy blood donors (donor T cells). Triplicate 10,000 T cells were seeded in round bottom 96-well plate in RPMI 10% AB serum either alone or mixed at 1:1 and 2:1 LCH T cell-to-donor T cell ratio and were stimulated with soluble OKT3 (50 ng/ml) in the presence of CD14^+^ cells (CD3:CD14 ratio is 1:5). The regulatory capacity of LCH T cells was based on the resulting T cell proliferation measured by the incorporation of (^3^H)-thymidine (3HTdR) per well added during the last 18 h of a 3- to 5-d culture. Cells were harvested using a Skatron harvester (Lier, Norway) and counted in a Tri-carb 2100TR liquid scintillation analyzer (Packard, http://www.packardbioscience.com/).

### T Cell Clonality

*T cell receptor gamma (TCRG)* rearrangement was analyzed on the DNA extracted from four frozen tumors. 100 ng of DNA was amplified in two different mixes using one set of three Jg fluorescent primers (Jg1/2–6FAM/JgP-HEX/JgP1/2-TET) and either VgfI/Vg10 primers or Vg9/Vg11 primers [30]. Identification of Vg and Jg utilization was, respectively, based on PCR product size and fluorescent aspect after Genescan analysis on a 310 automated DNA fragment analyzer (PE Biosystems, http://www.appliedbiosystems.com/). All PCRs were also analyzed by heteroduplex nondenaturing 8% polyacrylamide gel electrophoresis and ethidium bromide staining (EB PAGE). Cases were considered to be *TCRG* clonal if either multiplex reaction demonstrated at least one discrete band. This strategy provided a reproducible sensitivity of 1%–5%

### IL10 Transcript Quantification

DNA and RNA were successively extracted from frozen biopsies using AllPrep DNA/RNA mini kit (Qiagen, http://www.qiagen.com/). Cell number per biopsy was assessed in a real-time PCR assay by determining the number of beta-globin gene copies from extracted DNA (RTQ-PCR beta-globin kit, Roche, http://www.roche.com/). Complementary DNA was synthesized using random primers and Superscript-III. Interleukin (IL) 10 and 18S cDNA were amplified in the same run with IL10 forward primer (5′- GGCGCTGTCATCGATTTCTT-3′) and IL10 reverse primer (5′- GGCATTCTTCACCTGCTCCA-3′), or 18S forward primer (5′- AACGGCTACCACATCCAAGG-3′) and 18S reverse primer (5′- GGGAGTGGGTAATTTGCGC-3′) with Power SYBR Green PCR master mix using a 7300 Real Time PCR System (Applied Biosystems). cDNA from unstimulated or LPS-stimulated PBMCs of healthy donors were used as positive controls. The quantity of IL10 transcripts was calculated according to IL10 Ct and 18S Ct for each biopsy. Relative light units (RLUs) were determined by the IL10 biopsy-to-IL10 unstimulated PBMC ratio. Since beta-globin real-time PCR indicated an inconstant cell number per biopsy, RLUs were then adjusted to 10^4^ cells. The experiment was run twice with the same samples, and gave similar results.

## Results

### Active Neo-Angiogenesis, Tissue Remodeling, and T Cell Proliferation in LCH Granulomas

**Figure 1:**
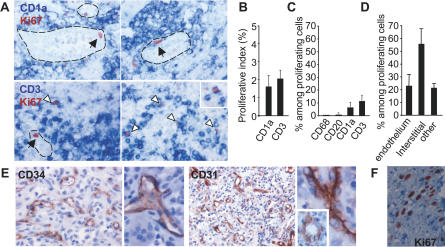
Proliferating Cells in LCH Granuloma are Mostly Endothelial Cells, Fibroblasts, and T Cells Paraffin-embedded and frozen sections were stained with antibodies against Ki-67 (which label proliferating cells), CD1a (LCs), CD3 (T cells), CD20 (B cells), CD68, CD31, and CD34 (endothelial cells). (A) Double immunostaining of paraffin-embedded section from LCH eosinophilic granulomas with anti-Ki-67 Ab, (brown nuclear staining) and with anti-CD1a Ab (upper images, blue staining) or anti-CD3 Ab (lower images, blue staining). Open arrowheads indicate double-stained cells, black arrowheads indicate Ki-67^+^ cells with an endothelial morphology. (B) Histogram represents percentage of CD1a^+^ cells and of CD3^+^ cells labeled with Ki-67 (*n* = 15). (C) Histogram represents percentage of proliferating cells (Ki-67^+^) that express CD1a, CD3, CD20, or CD68 (*n* = 15). (D) Histogram represents percentage of proliferating cells (Ki-67^+^) that are endothelial cells, interstitial cells (fibroblasts), and other types based on morphological examination. (E) Immunolabeling of blood vessels on paraffin-embedded section from LCH eosinophilic granulomas with CD34 (left) and CD31 (right) antibodies. (F) Proliferating Ki-67^+^ cells (brown nuclear staining) with a fibroblast-cell morphology in an eosinophilic granuloma.

The human Ki-67 protein is present in nuclei during all active phases of the cell cycle (late G1, S, G2, and mitosis) but is absent from resting cells (G0) [31]. Double staining for the detection of cell surface antigens and Ki-67 is an excellent tool to quantify the growth fraction of a given cell population in normal tissues and in tumors composed of mixed cell populations [32,33]. We performed immunostaining with antibodies against Ki-67, the LC antigen CD1a, CD68 (which is also expressed by macrophages), the T cell antigen CD3, and the B cell antigen CD20 in tissue samples from a retrospective series of 16 patients with various clinical forms of LCH (patients 1–12, 31, and 35–37; Table 1). We observed that a mean of 1.9% (range 0.8%–2.4%) of CD1a^+^ LCs were proliferating (Figure 1A and 1B). In the same specimens, a mean of 2% of CD3^+^ T cells were also proliferating (Figure 1A [open arrowheads] and 1B). CD1a^+^ cells accounted for only 6%, while CD3 T cells accounted for 12%, of proliferating cells (Figure 1C). B cells were present in some but not all cases and when present accounted for only 1% of proliferating cells. Proliferating cells did not stain for the macrophage marker CD68 (Figure 1C). Therefore, most (80%) of the proliferating cells in LCH tumors were negative for CD1a, CD3, CD68, and CD20 (Figure 1C). Examination of histological slides also clearly indicated that LCH granulomas were a site of extensive neo-angiogenesis and tissue remodeling, as evidenced by immunostaining using CD31 and CD34 antibodies (Figure 1D). We observed that 20% of Ki-67^+^ cells had the morphology and location of endothelial cells (Figure 1A [arrows] and 1E) and 50% were identified as fibroblastic cells (Figure 1F).

### Putative LC Precursors are Detected at Normal Levels in the Blood of LCH Patients

**Figure 2:**
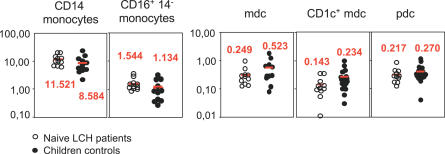
Putative LC Precursors are Detected at Normal Frequencies in the Peripheral Blood of Naïve LCH Patients CD14^+^ monocytes (DR^+^, CD14^+^, CD16^lo^) and CD16^+^ monocytes (DR^+^, CD14^−^, CD16^hi^), MDCs (DR^+^, CD11c^+^, lin^−^), CD1c^+^ MDCs (DR^+^, CD1c^+^, CD11c^+^, lin^−^), and PDCs (DR^+^, BDCA-2^+^, CD123^+^) from naïve (untreated) LCH patients and age-matched control children were studied by five-color flow cytometry. Data are represented as the percentage of PBMCs. *p*-Values (Kruskal-Wallis test) for the comparison between naïve (untreated) LCH patients and age-matched control children were not significant either in the number or in the frequency of DC and monocyte subsets present in the peripheral blood.

The low-growth fraction of CD1a^+^ LCs in LCH granulomas was similar in the different clinical forms of the disease (unpublished data). This observation indicates that proliferation of LCs is limited and may not account for granuloma maintenance and dissemination, and that other mechanisms may be involved such as the recruitment of LC precursors or increased LC survival. We therefore investigated whether putative LC precursors were increased in proportion, or in absolute numbers, in the blood of patients. Toward this aim we explored naïve patients at the time of diagnosis and before treatment (*n* = 12; patients 14–23, 25, and 29; see Table 1). Peripheral blood CD14^+^ monocytes, CD16^+^ monocytes, plasmacytoid DC (PDC), and classical MDC subsets were within the normal range in frequency (Figure 2) and numbers (unpublished data), in comparison to age-matched controls. Circulating CD1a^+^ cells were never detected in the peripheral blood of the patients or the control children (unpublished data). Together, these data suggest that LCs accumulate in granuloma by a mechanism other than local proliferation or recruitment of LC precursors. To investigate potential mechanisms that could lead to the increased survival of tumor LCs, we studied further the immunological characteristics of LCH granulomas.

### Accumulation of Polyclonal Regulatory CD4^+^ T Cells in LCH Granulomas

**Figure 3:**
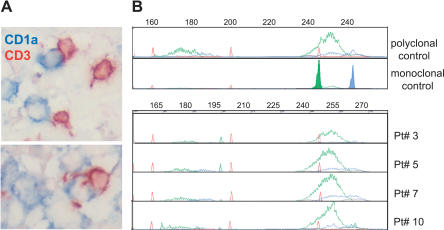
Polyclonal T Cells Infiltrate LCH Granuloma in Close Contact with LCs (A) Double immunohistochemical labeling of frozen sections from an eosinophilic granuloma with anti-CD3 (brown) and anti-CD1a antibodies (blue). (B) T cell receptor gamma rearrangement was determined on the DNA extracted from frozen biopsies from four patients. Fluorescent profiles for Vgfl/Vg10 PCR using fluorescent Jg primers (JgP, red; Jg1/2, green; JgP1/2, blue) are shown; all the biopsies display a polyclonal profile in comparison to polyclonal and monoclonal positive controls.

**Figure 4:**
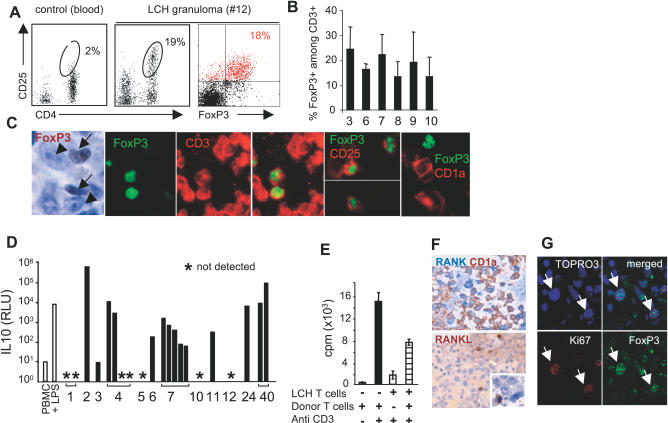
CD4^+^ CD25^+^ FoxP3^high^ T Cells Accumulate in LCH Granulomas (A) Identification of CD4^+^ CD25^+^ FoxP3^+^ T cells by flow cytometry on fresh granuloma tissue. Dot plots are representative of similar data obtained in three patients (11, 12, and 29). (B) Histograms represent percentage of FoxP3^+^ T cells among CD3^+^ T cells in eosinophilic granuloma: 17.2% ± 3.36% of T lymphocytes expressed FoxP3 (*n* = 6). (C) Left image, immunohistochemical staining on a paraffin-embedded section with FoxP3 antibodies showing FoxP3^+^ T cell (arrow, brown nucleus) in close contact with histiocytes (arrowhead). Right images, double immunofluorescence labelling with anti-FoxP3 (green nucleus) and anti-CD3^+^, anti-CD25^+^, or anti-CD1a (red) in frozen sections. (D) Real-time PCR assay showing a high level of IL10 transcripts in eosinophilic granulomas (*n* = 12 patients). (E) T cells were isolated by CD3^+^ selection from a fresh LCH biopsy (patient 12; LCH T cells) and from the peripheral blood of a healthy blood donor (Donor T cells) and were stimulated by soluble OKT3 (Anti-CD3) in the presence of CD14^+^ monocytes. Unsorted T cells from an LCH granuloma (LCH T cells) caused an approximate 50% decrease in the proliferation of normal T cells. Filled bar, healthy donor T cells alone, open bar LCH T cells alone, hatched bar, mix of healthy donor T cells and LCH T cells. (F) Immunohistochemistry on paraffin-embedded section from an eosinophilic granuloma using RANK (upper) and RANKL antibodies (bottom) (*n* = 9 patients). (G) Double immunolabeling with Ki-67 and FoxP3 antibodies of frozen sections from an eosinophilic granuloma (*n* = 2 patients).

CD3^+^ T cells were often found in intimate contact with CD1a^+^ LCs (Figure 3A). We therefore investigated their phenotype, clonal origin, and function. The existence of clonal populations among T cells was investigated by exploring *TCRG* rearrangement with a multiplex fluorescent PCR with a sensitivity of 5 × 10^−2^ [30]. T cells that accumulated in the LCH lesions were polyclonal (Figure 3B), in line with previous results [34]. In tissue samples available for this analysis, we observed that 13%–25% of T cells (*n* = 11; mean ± SD, 18% ± 7%) of CD3^+^ T cells coexpressed CD4^+^, CD25^hi^, and strongly expressed the transcription factor FoxP3, as determined by flow cytometry (patients 11, 12, and 29) and by in situ immunofluorescence staining (patients 3 and 6–10) (Figure 4A–4C). FoxP3^+^ T cells were located within LCH granulomas, in close contact with histiocytes and with FoxP3^−^ lymphocytes (Figure 4C). In line with the presence of T-regs, transcripts for the cytokine IL10 were abundant in 14 samples from eight patients (patients 2, 3, 4, 6, 7, 11, 24, and 40) out of 12 available for this analysis (Figure 4D). In addition, T cells could be purified from the biopsy of patient 12, and were incubated with allogenic peripheral blood T cells, monocytes, and anti-CD3 antibody (Figure 4E). These granuloma-derived T cells inhibited allogenic T cell proliferation at a 1:1 ratio, albeit by only 50%, suggesting that they indeed exert a regulatory activity. Of note, FoxP3^+^ T cells were scarce (4.07% ± 1.47% of CD3^+^ T cells) in lesions from four children with SHML, a non-Langerhans cell histiocytosis that features the accumulation of CD1a^−^/CD68^+^ histiocytes.

### Expansion of T-Regs in Patients with LCH

**Figure 5:**
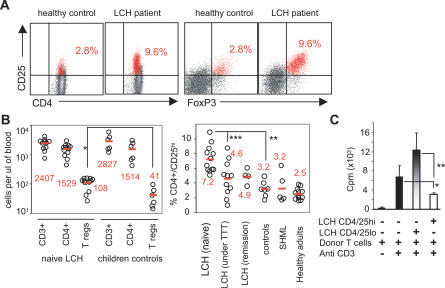
Regulatory CD4^+^ CD25^+^ FoxP3^+^ T Cells Are Expanded in the Peripheral Blood of LCH Patients (A and B) Flow cytometry analysis of CD4^+^/CD25^hi^ FoxP3^high^ cells in the blood of LCH patients in comparison to healthy controls. (A) Representative dot plots from the blood of one patient and one control. (B) CD3 and CD4 lymphocyte counts were similar in naïve (untreated) LCH patients at diagnosis and in control children, but the absolute number and the frequency of T-regs were significantly higher in the blood of naïve LCH patients at diagnosis than in patients with LCH under treatment, in control children, and in children with SHML. *Chi-squared = 7.125 with 1 df, *p* = 0.0076; **Chi-squared = 13.714 with 1 df, *p* < 0.001; ***Chi-squared = 6.682 with 1 df, *p* = 0.0097. (C) Regulatory activity of CD3^+^ CD4^+^ CD25^hi^ T cells isolated from the blood of LCH patients. Proliferation of control T cells (Donor T cells) induced by soluble OKT3 (Anti CD3) in the presence of CD14^+^ monocytes was inhibited by the addition of sorted CD3^+^ CD4^+^ CD25^hi^ T cells (LCH CD4/CD25hi) isolated by positive selection from the blood of a patient with LCH, but not by CD3^+^ CD4^+^ CD25^low^ T cells (LCH CD4/25lo) isolated from the blood of the same patient. *p < 0.02, **p = 0.01.

RANK and RANKL have been found to be involved in the systemic increase of T-regs by RANK-expressing skin DCs [35]. We found that LCs from LCH granulomas expressed RANK (Figure 4F; *n* = 9; patients 1, 3, 4, 7, 9, 29, 31, 35, and 36), as previously described [36]. RANKL was also expressed in LCH granulomas; however, in contrast to a previous report [35], RANKL appeared to be expressed by small lymphocytes rather than LCH cells (Figure 4F). Ki-67 protein was detected in the nuclei of FoxP3^+^ T cells, suggesting that they were proliferating (Figure 4G: *n* = 2; patients 6 and 7). To further investigate whether T-regs actually expand in LCH patients or accumulate within LCH granuloma, we examined the number of peripheral blood T-regs in untreated, “naïve” patients with newly diagnosed LCH (*n* = 12, patients 14–23, 25, and 29; see Table 1), patients with active disease under treatment or after treatment (*n* = 10; patients 12, 13, 16, 20, 24, 26, 27, and 30–32), or patients in clinical remission (*n* = 4; patients 3, 28, 33, 34; Table 1) in comparison with samples from adult and children controls matched for age with LCH patients, and from children with SHML. Naïve LCH patients had, overall, normal blood lymphocyte counts, but their absolute number and the frequency of blood CD3^+^ CD4^+^ CD25^hi^ FoxP3^high^ T cells were significantly higher at diagnosis than in age-matched controls and children with SHML (Figure 5A and 5B). Sorted CD4^+^/CD25^hi^ T cells, but not CD4^+^/CD25^lo^ T cells, from one LCH patient out of one tested (patient 29), inhibited CD3-induced proliferation of a healthy donor T cells (Figure 5C). The higher number and frequency of T-regs in the peripheral blood of children with LCH in comparison to controls suggests that T-regs expand in patients with LCH. In addition, the observation of Ki-67^+^ T-regs and RANK/RANKL expression in the granuloma (see Figure 4F and 4G), suggests that the expansion of T-regs takes place at least in part in the granuloma. We found that children with LCH during and after treatment had a decreased frequency of blood CD3^+^ CD4^+^ CD25^hi^ FoxP3^high^ cells (Figure 5B). This finding suggests that the increased frequency of CD4^+^ CD25^hi^ FoxP3^high^ cells in LCH patients correlated with active disease. Therefore, patients with LCH have an expanded FoxP3^high^ T-reg population in the blood, which could be relevant to the pathophysiology of the disease.

### Impaired Delayed-Type Hypersensitivity Response in Patients with LCH

Patients with LCH do not exhibit overt immunodeficiency. However, CD4^+^ CD25^high^ FoxP3^high^ T-regs have been shown to inhibit T cell activation, in an antigen-independent manner [37,38] and can inhibit cellular immune response such as delayed type hypersensitivity (DTH) to tuberculin [39]. We therefore investigated whether patients with LCH had a normal DTH response to tuberculin. DTH responses to tuberculin were evaluated in seven children previously vaccinated with BCG. All seven patients had negative DTH test to PPD (Table 2). Information about previous DTH responses to tuberculin before the onset of LCH was available for five patients, of whom three had tested positive and two negative. The frequency of negative DTH responses to tuberculin (PPD) after vaccination with BCG ranges from 10% up to 30% in France [29,39]. In this latter hypothesis, the probability of observing seven negative DTH responses among seven tested patients, according to Bernouilli's distribution, is very low (*p* = 2.2 × 10^−4^). Despite the small number of patients, our observations favor the hypothesis that LCH is associated with impaired DTH.

## Discussion

The results from the present study indicate that fibroblasts, endothelial cells, and T cells account for the bulk of proliferating cells in LCH granulomas. This result is in line with the pathological description of the disease as a granuloma. LCs themselves exhibited a proliferation index of only 1.9%, and represented only 6% of proliferating granuloma cells. Putative LC precursors such as blood monocytes and DCs were not expanded in the blood of patients. Therefore, the accumulation of LCs in granulomas is mainly the consequence of increased survival rather than proliferation.

An explanation for increased survival of LCs is our finding that LCH is associated with the local and systemic expansion of T-regs, which may in turn impair the resolution of chronic granulomas. Local proliferation of LCs may thus contribute relatively little to granuloma maintenance and dissemination compared to the effect of immunological mechanisms. Although previous studies have reported high cell proliferation within LCH granulomas [3,18,19], the conclusion that LC-type cells were proliferating was not directly supported by these studies, because granulomas are a mix of several cell types. Our results indicate that neo-angiogenesis and tissue remodeling account for a substantial proportion (80%) of proliferating cells, while the proliferative index of LCs is low, on average 1.9%. A recent study has found moderately increased frequencies of MDCs in the blood of a fraction of patients with LCH [9]. In a previous study by Hosmalin et al. [40] eight out of ten patients had normal frequencies of blood DCs, while two patients with severe disease had an increased frequency of circulating DCs. In the studies of Rolland et al. [9] and Hosmalin et al. [40], some patients have been previously treated by steroids and/or cytotoxic chemotherapy, and it is thus difficult to ascertain whether the increased frequency of MDCs in these patients was due to the disease or to the treatment, because the numbers and relative frequencies of myeloid cells in the blood can be affected by steroids and cytotoxic chemotherapy.

In the present prospective study, patients were analyzed at the time of diagnosis and before any treatment, and we did not observe a significant increase in either number or frequency of monocytes or blood MDCs in 11 out of 11 patients. Therefore, increased frequency of blood MDCs is not a general feature of LCH.

The present data do not rule out a neoplastic origin of LCH lesions; however, we propose that enhanced cell survival, rather than uncontrolled LC proliferation as occurs in neoplasia, is likely to play a major role in the maintenance and dissemination of these slow-growing tumors. A potential involvement of immune mechanisms in the pathogenesis of LCH is probable, because DCs are key regulators of the immune response [41], both initiating adaptive immunity and inducing unresponsiveness or tolerance [42,43]. The regulatory function of T-regs in granulomas and blood of patients with LCH was suggested by their phenotype (CD4^+^ CD25^high^ FoxP3^high^), their intimate contact with LCH histiocytes (Figure 3A), their ability to inhibit T cell proliferation in vitro (see Figures 4B and 5C), the presence of IL10 transcripts in the lesion, and the lack of DTH response in vivo in patients with LCH (see Table 2). Another limitation of this study is that the functional data were obtained on a small number of patients, due to the rarity of the disease and the extreme difficulty of purifying cell populations from small, rare biopsies. However, altogether these data provide an explanation for the paradox of an “antigen-presenting cell tumor” that does not induce its own rejection by the immune system.

LC-type cells in LCH granulomas have been found to be in an immature, or semimature, stage of differentiation in vivo and to only weakly stimulate allogeneic T cell proliferation [5]. Immature and/or semimature DCs are prone to induce T-regs that inhibit polyclonal T cell responses and promote tolerance [43–46], whereas mature DCs stimulate effector T cells, facilitating immunity [43]. Therefore we have proposed that the accumulation of immature LCs in LCH granulomas may inhibit the efficient immune response against these LCs [5]. The idea that LCs may play a role in the down-regulation of the cutaneous immune response is supported by three studies; first, a study in humans showed that immature LCs accumulate in skin-draining lymph nodes in patients with cutaneous infections or malignancies in the absence of autoimmune response such as vitiligo [47]; second, a recent work suggested that mice without LCs have increased skin contact hypersensitivity [48]; a third study showed that environmental stimuli at the skin can redirect the local and systemic immune system by means of RANKL [35]. In addition, IL10 has been repeatedly detected in T cells and macrophages within LCH granulomas [5,49] and has been shown to play a role in locking immature DCs in a tolerogenic state, which in turn induces T-regs that produce more IL10 [50,51]. Thus a positive feedback loop might ensure prolonged immunosuppression within LCH granulomas. Indeed, recent studies have shown in vivo that T-regs in contact with DCs resulted in the inhibition of subsequent T cell activation by these DCs, supporting the idea that DCs are central to T-reg function in vivo [52].

One hypothesis for LCH pathogenesis is, therefore, that T-regs accumulate in contact with immature LCs and inhibit the immune response against these LCs, leading to increased survival and granuloma maintenance and dissemination. T-regs are polyclonal in LCH lesions and their expansion is therefore likely to be a consequence of the disease. Increased knowledge of the pathophysiology of LCH and LCs is important for better management of the disease and for the benefit of the patients. The finding that LCH cells may be involved in the expansion of T-regs allows a different and novel view on the pathogenesis of this disease, and may lead to the development of new, hitherto unconsidered, therapeutic strategies in LCH. Drugs that target T-regs, or overcome tolerance, may be beneficial to some patients. In return, LCH may also teach us important information about the biology and functions of normal Langerhans cells in vivo.

## Abbreviations

DC: dendritic cell
DTH: delayed type hypersensitivity
IL: interleukin
LC: Langerhans cells
LCH: Langerhans cell histiocytosis
MDC: myeloid dendritic cell
PDC: plasmacytoid dendritic cell
PBMC: peripheral blood mononuclear cell
SHML: sinus hyperplasia with massive lymphadenopathy
TCR: T cell receptor
T-reg: regulatory T cell
